# Down Regulation of CLDND1 Induces Apoptosis in Breast Cancer Cells

**DOI:** 10.1371/journal.pone.0130300

**Published:** 2015-06-17

**Authors:** Chandrani Achari, Sofia Winslow, Christer Larsson

**Affiliations:** Translational Cancer Research, Department of Laboratory Medicine, Lund University, Lund, Sweden; Emory University, UNITED STATES

## Abstract

Identification of targets for apoptosis induction is important to provide novel therapeutic approaches in breast cancer. Our earlier studies showed that down regulation of protein kinase C δ (PKCδ) induces death in breast cancer cells. In this study we set out to identify previously unrecognized apoptosis regulators in breast cancer cells. To identify candidates, global expression analysis with microarray was performed after down regulation of PKCδ in the basal-like breast cancer cell lines MDA-MB-231, MDA-MB-468 and BT-549. Genes that were down regulated in all cell lines were further studied for survival-supporting effects. The claudin-like CLDND1 was singled out since several independent siRNAs targeting CLDND1 induced cell death in several cell lines. The cell death induced by CLDND1 knockdown was caspase-dependent, suggesting induction of apoptosis. Nuclear fragmentation, cleavage of caspase-3 and PARP and release of cytochrome C from the mitochondria upon CLDND1 depletion demonstrated involvement of the intrinsic apoptotic pathway. Inhibition of MEK1/2 and JNK further potentiated the cell death induction by CLDND1 knockdown. However, CLDND1 down regulation augmented ERK1/2 phosphorylation, which thereby may protect against the apoptosis inducing effects of CLDND1 down regulation. A concomitant inhibition of MEK1/2 suppresses the ERK1/2 phosphorylation and markedly potentiates the cell death following CLDND1 siRNA treatment. There is today little information on the function of CLDND1. These data provide novel information on CLDND1 and highlight it as a novel survival factor in basal-like breast cancer cell lines.

## Introduction

Breast cancer is the most frequently diagnosed cancer among women worldwide and despite significant advances towards targeted therapy and screening techniques, breast cancer continues to be the major cause of cancer-related deaths [1]. Both abnormal proliferation and failure to activate apoptosis are major contributors leading to malignant cellular transformation [2, 3]. Identification of signal transduction targets for apoptosis induction is therefore of importance to provide novel opportunities for therapeutic approaches. For breast cancer, there are several potential signaling pathways that can be targeted to remove the survival support.

Earlier we showed that down regulation of protein kinase C δ (PKCδ) induces death in breast cancer cells [4]. The onset of cell death is rather slow and we have therefore hypothesized that it requires novel protein synthesis. In this study we set out to identify one or several of these potential apoptosis regulators in breast cancer cells. Three different breast cancer cell lines were treated with a PKCδ siRNA to induce cell death. Global expression analysis was performed and genes that were consistently up or down regulated were identified. One gene that was altered in all cells and also was seen to support survival in all cells was *CLDND1* which encodes a protein that has been denoted claudin-25 [5] and belongs to a protein family which encompasses claudins.

The role of claudins in carcinogenesis and progression to metastasis is an active area of investigation as a result of the frequent finding of altered claudin expression in several cancers. Claudins belong to the family of tight junction proteins that play an important role in the regulation of paracellular permeability and maintaining cell polarity in epithelial and endothelial cell sheets [6]. They are also vital for cell-cell interaction and for the maintenance of differentiated state of epithelial cells [7, 8]. Claudins are 21–28-kD transmembrane proteins having four transmembrane helices with their amino- and carboxy-terminal tails extending into the cytoplasm [9]. They constitute 26 family members in humans [10] and their expression appears to be tissue-specific [11, 12]. The claudins are capable of recruiting signaling proteins, thereby regulating various cellular processes including cell proliferation, differentiation and tumorigenesis [13–15]. Claudins are deregulated in a variety of malignancies [16–18]. In some studies claudin-3 and -4 are overexpressed in breast cancer and in contrast, claudin-1 and claudin-7 are down regulated, or, completely absent [19, 20]. Reduction in claudin-16 has been linked to aggressive tumors and high mortality in human breast cancer patients [21]. Increased expression of claudin-1 and -4 is associated with basal-like breast cancer subtype, which is often related to poorer outcomes [22]. Moreover, increases in claudin-4 correlated to adverse outcome including patients that have received adjuvant tamoxifen [23]. In addition, low levels of claudin-3, -4, and -7 is a hallmark of a subgroup of mainly triple-negative (no amplification of *HER2* and negative for estrogen and progesterone receptors) breast cancers with mesenchymal and cancer stem cell-like features [24, 25].

Claudin domain containing protein 1 (CLDND1), a claudin-like protein also known as claudin-25 [5, 10] is a multi-pass membrane protein that belongs to the PMP-22/EMP/MP20 family. It is widely distributed in the adult CNS with highest expression in the corpus callosum, caudate nucleus, cerebral cortex, medulla, putamen, spinal cord, substantia nigra and subthalamic nucleus and weakly in the adult heart [26]. However, not much is known about the function of CLDND1. In this study we demonstrate that it may play a role as a survival factor in breast cancer.

## Materials and Methods

### Cell Culture

All cell lines were obtained from American Type Culture Collection. MDA-MB-231, MDA-MB-468, BT-549 and MCF-7 breast cancer cells were maintained in RPMI 1640 medium (HyClone, Thermo Scientific) supplemented with 10% fetal bovine serum (EuroClone), 1 mM sodium pyruvate (HyClone, Thermo Scientific) and 100 IU/ml penicillin-streptomycin solution (HyClone, Thermo Scientific). The medium for BT-549 cells was additionally supplemented with 0.01 mg/ml insulin (Novo Nordisk A/S).

### Transfections

For siRNA transfections, cells were seeded at 40–50% confluency and grown in complete medium without antibiotics for 24 h. Cells were thereafter transfected for 72 h using 2 μl/ml Lipofectamine 2000 (Invitrogen) and 40 nM siRNA (Invitrogen) (Table 1) in Opti-MEM I (Invitrogen) according to the supplier’s protocol. Where indicated, benzyloxycarbonyl-Val-Ala-Asp-(*O*methyl) fluoromethyl ketone (Z-VAD-fmk; 20 μM), PD98059 (35 μM), SB203580 (10 μM), SP600125 (20 μM) (all from Sigma), U0126 (20 μM) (from Cell Signalling) Wortmannin (100 nM), or an equal volume of dimethyl sulfoxide was present from the initiation of transfection or for 24h.

**Table 1:** siRNA oligo nucleotides.

siRNA oligonucleotide	Sequence
Control	
CLDND1-1	GAAACCACAAUAGCGGGAUUGAUCU
CLDND1-2	UGUGCUUGCAUUUGCCGAAGCUUAU
CLDND1-3	CAGUAAGUUGUUAUGUUGCUGGAAU
PLEKHA2	CCCAGGCUAACUGCCUCCUCUGGUA
ACSL3	GGAGAGGAAGAUGUCUACAUUGGAU
SLC30A9	CCAGAUCCUUCUCAUCCGUACGGAU
MSN	GCUGAGCUGAAGACUGCCAUGAGUA
CALM3-1	CCACUGAAGCAGAGCUGCAGGAUAU
CALM3-2	GAGGUGGAUGCAGAUGGGAACGGGA
CALM3-3	GAUGAUCAGGGAGGCUGACAUCGAU
PRKAR1A	GGACCGACCUAGAUUUGAACGUGUU

### RNA extraction and expression analysis

Total RNA was extracted from MDA-MB-468 and BT-549 cells following PKCδ knockdown, with the RNeasy kit (Qiagen). The RNA samples were then treated with RQ1 RNAse-free DNase (Promega) to eliminate potential DNA contamination and evaluated on a Bioanalyzer 2100 (Agilent). RNA was amplified and labeled with Illumina TotalPrep-96 RNA Amplification Kit. Labeled RNA was hybridized to the Illumina Human HT-12 v4 Expression BeadChip. Data were normalized using quantal normalization. The data for MDA-MB-468 and BT-549 cells are deposited in GEO (Accession number GSE 55503). MDA-MB-231 data have been published and are deposited in Array Express (Accession E-MTAB-462). Differential gene expression was analyzed with the limma package in R.

### Western blot analysis

Cells were lysed in buffer (10 mM Tris-HCl, pH 7.2, 160 mM NaCl, 1% Triton X-100, 1% sodium deoxycholate, 0.1% SDS, 1 mM EDTA, and 1 mM EGTA) containing 40 μl/ml Complete protease inhibitor (Roche Applied Science) and incubated on ice for 30 min. Lysates were cleared by centrifugation at 14,000 x *g* for 10 min at 4°C, diluted in sample buffer, and heated at 105°C for 5 min. Protein concentration was determined by Bradford assay. Equal amount of proteins were electrophoretically separated on either 10% or 12% NuPAGE Novex BisTris gels (Invitrogen) and transferred to polyvinylidene difluoride membranes (Millipore). Membranes were blocked with PBS containing 5% nonfat milk and probed with antibodies to caspase-3, phospho-ERK1/2 and ERK1/2, (1:500; Cell Signaling); PARP (1:500; BD Biosciences), cytochrome c (1:500; BD Biosciences) and actin (1:2000; MP Biomedicals). Proteins were visualized with horseradish peroxidase-labeled secondary antibody (Amersham Biosciences) using the SuperSignal system (Pierce) as substrate. Chemiluminescence was detected with a CCD camera (Fujifilm).

### Analysis of Viable Cell Number

Cells were seeded at a density of 2000 cells/well in 96-well culture plates and transfected with siRNA for 72 h. The amount of viable cells was assessed by a WST-1 cell viability assay (Roche Applied Science). Absorbance was measured in an Anthos 2020 enzyme-linked immunosorbent assay plate reader (Anthos Labtec Instruments).

### Annexin V Analysis

Following transfection, floating cells, pooled with trypsinized adherent cells, were stained with Annexin V-allophycocyanin (APC; Pharmingen) according to the supplier’s protocol, and the amount of bound Annexin V-APC was quantified with a FACSCalibur cytometer (BD Biosciences). 10,000 events were acquired on the FL-4 channel for the Annexin V-APC signal.

### Nuclear Morphology Analysis

MDA-MB-468 cells were seeded on glass coverslips at a density of 150,000 cells/35-mm cell culture dish and transfected with siRNA for 72 h. Following transfections, cells were fixed in 4% paraformaldehyde, washed with PBS and mounted on glass slides with a drop of Vectashield mounting medium with DAPI (Vector Labs) and nuclei were examined with fluorescence microscopy.

### Real-time qPCR

Total RNA was extracted with the RNeasy kit (Qiagen), and potential DNA contamination was eliminated with the RQ1 RNAse-Free DNase (Promega). Two micrograms of total RNA was used for cDNA synthesis with MultiScribe Reverse Transcriptase (Applied Biosystems). The cDNA was thereafter analyzed with Applied Biosystems 7300 real-time quantitative PCR system using the SYBR Green PCR Master Mix (Applied Biosystems). All primer pairs (Table 2) were designed according to RTPrimerDB or by using the Primer Express software (Applied Biosystems). The mRNA expression data were normalized to three reference genes (SDHA, UBC and YWHAZ). For relative quantification of gene expression, the comparative Ct method was applied.

**Table 2:** qPCR primers.

Primers for qPCR	Sequence 5′to 3′
**SDHA** forward	TGGGAACAAGAGGGCATCTG
**SDHA** reverse	CCACCACTGCATCAAATTCATG
**YWHAZ** forward	ACTTTTGGTACATTGTGGCTTCAA
**YWHAZ** reverse	CCGCCAGGACAAACCAGTAT
**UBC** forward	ATTTGGGTCGCGGTTCTTG
**UBC** reverse	TGCCTTGACATTCTCGATGGT
**CLDND1** forward	TGGTATAGCCCACCAGAAAGGAC
**CLDND1** reverse	CAATCCCGCTATTGTGGTTTCCG
**CALM3** forward	AATCTCTTCTCATCCACCCTCT
**CALM3** reverse	TCACGCCTACCTGCCCTTAC

### Subcellular fractionation

MDA-MB-468 cells were washed twice in ice-cold PBS, re-suspended in buffer [250 mM sucrose, 20 mM HEPES/KOH (pH 7.4), 10 mM KCl, 1.5 mM EGTA, 1.5 mM EDTA, 1 mM MgCl_2_ 1 mM DTT, digitonin C_E_ = 0.05%) and 40 μl/ml complete protease inhibitor (Roche Applied Science)] and homogenized with dounce homogenizer. Homogenates were centrifuged for 5 min at 800xg and supernatants were further centrifuged at 22000xg for 15 min. Supernatants were used as a cytosolic mithochondria-free fraction and the pellet was extracted and used as the membrane fraction containing mitochondria.

### Statistical Analysis

ANOVA followed by Duncan’s multiple range test was used for statistical analysis. The difference was considered significant if the p-value was < 0.05. All statistical calculations were performed using SPSS V.11.0.

### Accession Numbers

The data for MDA-MB-468 and BT-549 cells are deposited in GEO (Accession number GSE 55503). MDA-MB-231 data have been published and are deposited in Array Express (Accession E-MTAB-462).

## Results

### Effects of siRNAs targeting candidate survival genes on breast cancer cell death

**Fig 1:**
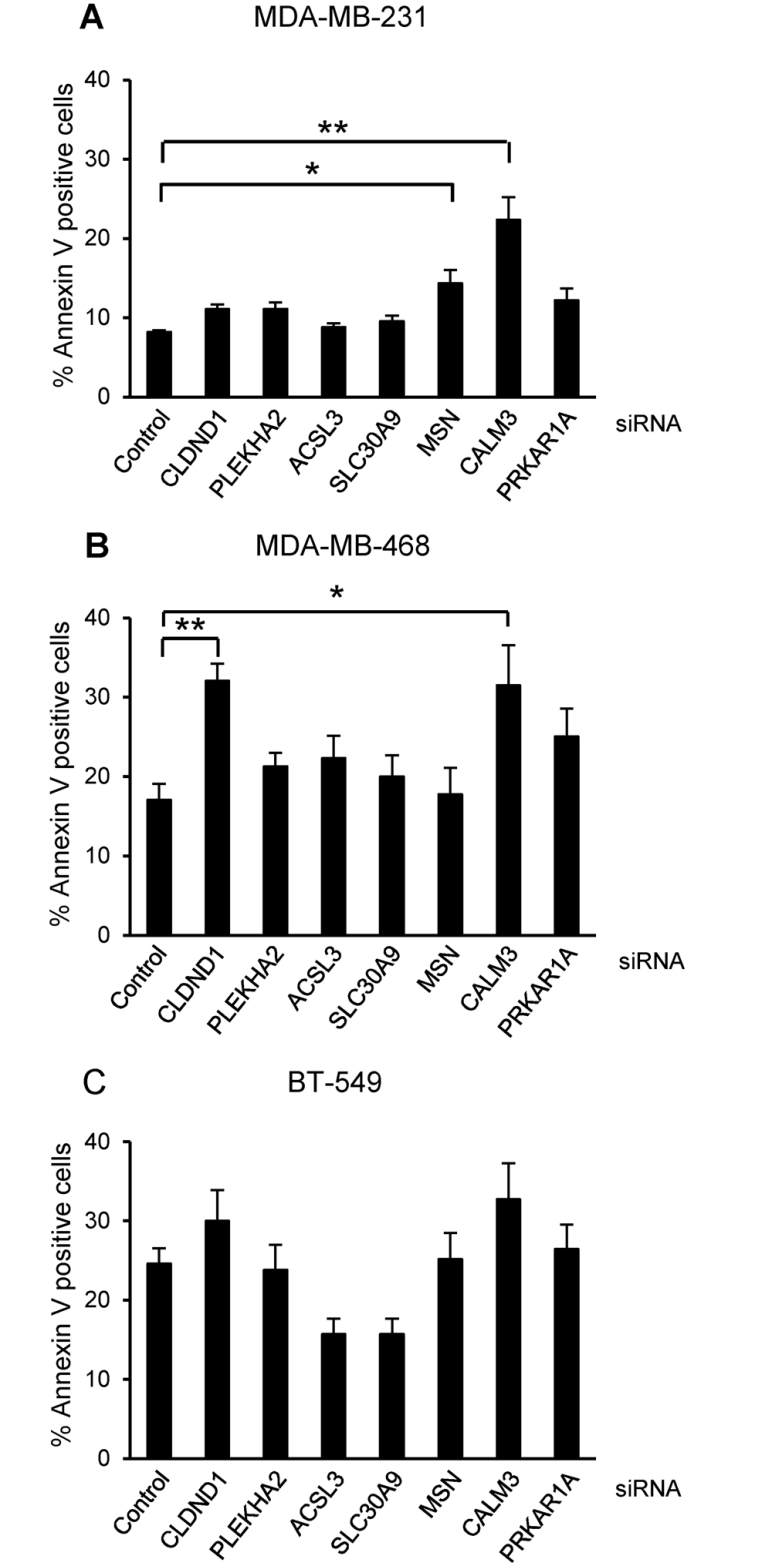
Effects of silencing CLDND1, PLEKHA2, ACSL3, SLC30A9, MSN, CALM3 and PRKAR1A on breast cancer cell death. MDA-MB-231 (A), MDA-MB-468 (B) and BT-549 (C) breast cancer cells were transfected with siRNA oligonucleotides targeting CLDND1, PLEKHA2, ACSL3, SLC30A9, MSN, CALM3, PRKAR1A or with a control siRNA for 72 h. Cells were subjected to Annexin V-APC staining and flow cytometry analysis. Data (mean ± S.E.M., n = 3) represent percent Annexin V-positive cells related to control conditions. Asterisks indicate statistically significant differences (*, p<0.05; **, p<0.01) compared with control cells.

To identify cell death regulators of breast cancer cell death, apoptosis was induced by treatment with a PKCδ siRNA, as previously shown [4], and the global gene expression was analyzed. Analysis of differential expression levels highlighted *CLDND1*, *PLEKHA2*, *ACSL3*, *SLC30A9*, *MSN*, *CALM3* and *PRKAR1A* as potential regulators of breast cancer cell survival since they were affected by PKCδ siRNA in all cell lines (Table 3). To study their potential role in cell death the breast cancer cell lines were treated with siRNAs targeting the genes (Fig 1). CALM3 siRNA effectively increased cell death, measured as Annexin V positivity, in both MDA-MB-231 and MDA-MB-468 cells and had a similar tendency in BT-549 cells. The siRNA targeting CLDND1 also induced cell death in MDA-MB-468 cells and had a similar tendency in MDA-MB-231 and BT-549 cells. The siRNAs targeting the other candidate genes had less consistent cell-death inducing effects (Fig 1). Based on these data it is premature to conclusively exclude a role in cell survival for these genes. However, based on this initial screening CALM3 and CLDND1 were selected as the most promising candidates for further study.

**Table 3:** Genes influenced by PKCδ siRNA in all cell lines. FC indicates change in log2 value between cells treated with siPKCδ and a control siRNA and is the mean of three experiments. This value, as well as the adjusted p-value, was obtained from analyses with the limma package in R.

Gene	BT-549		MDA-MB-231		MDA-MB-468	
	FC	Adj p-val	FC	Adj p-val	FC	Adj p-val
ACSL3	-1.51	1.12e-05	-1.34	1.01e-05	-1.75	1.72e-06
CALM3	-1.19	7.09e-05	-1.05	2.11e-05	-1.17	6.41e-05
CLDND1	-0.94	1.01e-05	-1.13	1.95e-05	-1.32	2.43e-07
MSN	-1.40	8.81e-06	-1.36	4.46e-06	-1.96	2.10e-07
PLEKHA2	-0.99	1.23e-04	-1.87	7.33e-06	-1.34	8.58e-06
PRKAR1	-1.68	1.11e-06	-1.31	8.91e-06	-1.90	1.08e-07
SLC30A9	-0.89	6.00e-05	-1.19	9.48e-06	-1.16	4.98e-06

### CLDND1 depletion decreases viability of different breast cancer cell lines

As an initial step we sought to exclude off-target effects of siRNAs by evaluating two additional siRNAs for both CALM3 and CLDND1. In these experiments CALM3 was excluded since none of the two additional siRNAs led to cell death (not shown) and we therefore continued with CLDND1.

**Fig 2:**
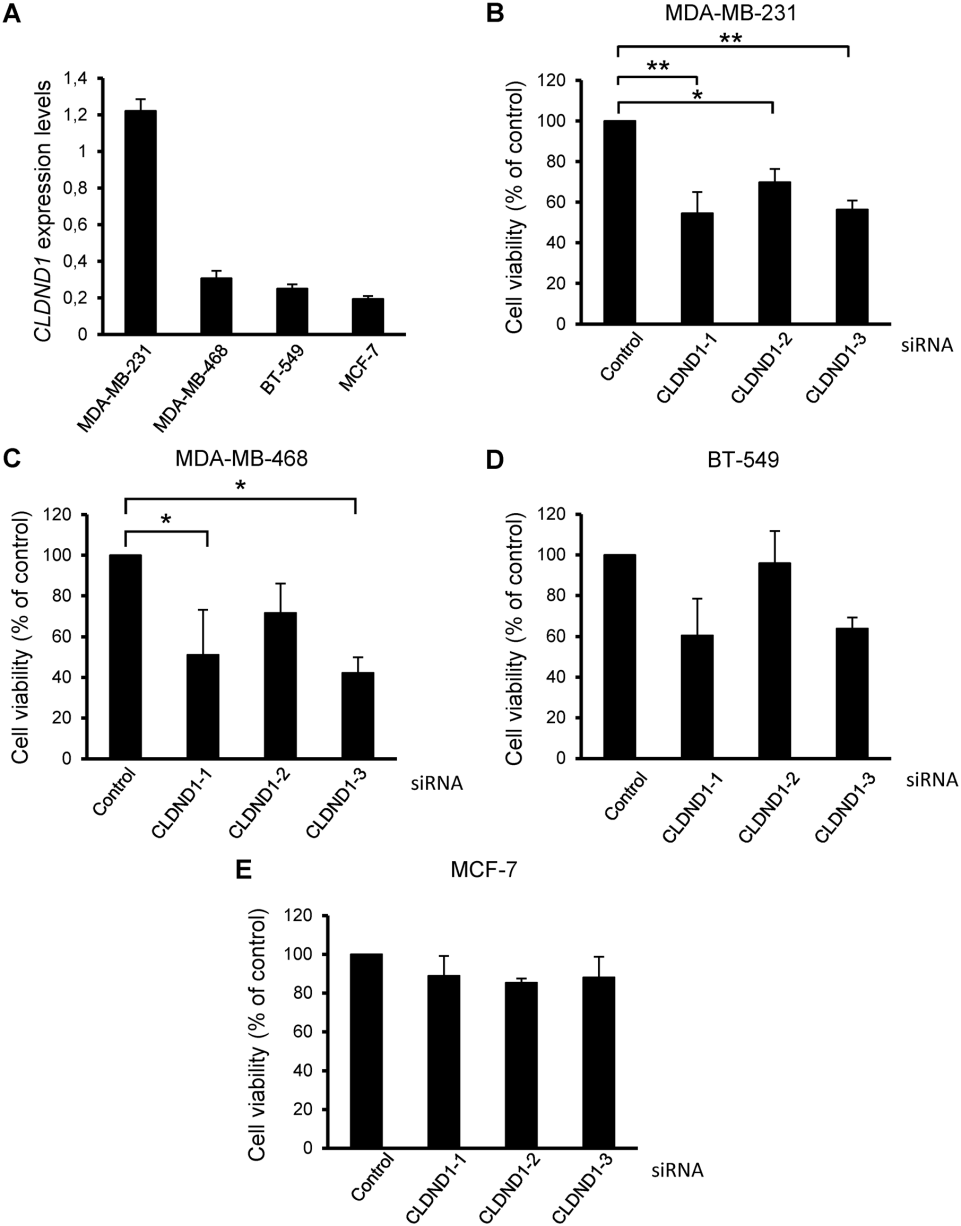
CLDND1 depletion decreases viability of different breast cancer cell lines. (A) mRNA expression of CLDND1 in various breast cancer cell lines. MDA-MB-231, MDA-MB-468, BT-549 and MCF-7 cells were incubated under normal conditions for 24 h. Cells were subsequently harvested and were analyzed for mRNA expression of CLDND1 with real-time quantitative PCR. In B-E, MDA-MB-231, MDA-MB-468, BT-549 and MCF-7 cells respectively, were transfected with three different siRNA oligonucleotides targeting CLDND1 or with a control siRNA for 72 h. Cells were thereafter subjected to WST-1 assay. Data (mean ± S.E.M., n = 3) represent the amount of viable cells expressed as percent viable cells obtained under control conditions (B-E). Asterisks indicate statistically significant differences (*, p<0.05; **, p<0.01) compared with control cells.

The role of CLDND1 in breast cancer cells was validated by transfecting MDA-MB-231, MDA-MB-468, BT-549 and MCF-7 cells with three different siRNA oligonucleotides targeting CLDND1 (Fig 2B–2E). A marked reduction in cell viability, as measured by a WST-1 assay, was detected following treatment with all CLDND1 siRNAs in MDA-MB-231 cells (p<0.05 for siCLDND1-2 and p<0.01 for siCLDND1-1 and siCLDND1-3). For MDA-MB-468 and BT-549 cells less pronounced effects, but with the same tendency, could be seen (Fig 2B–2D). However none of three CLDND1 siRNAs had any substantial effect on MCF-7 cells (Fig 2E). We also measured the basal levels of CLDND1 mRNA in MDA-MB-231, MDA-MB-468, BT-549 and MCF-7 cells by doing qPCR analysis of CLDND1 gene (Fig 2A). Expression of CLDND1 was much higher in MDA-MB-231 cells compared to MDA-MB-468, BT-549 and MCF-7 cells.

### Down-regulation of CLDND1 induces death in several breast cancer cell lines

**Fig 3:**
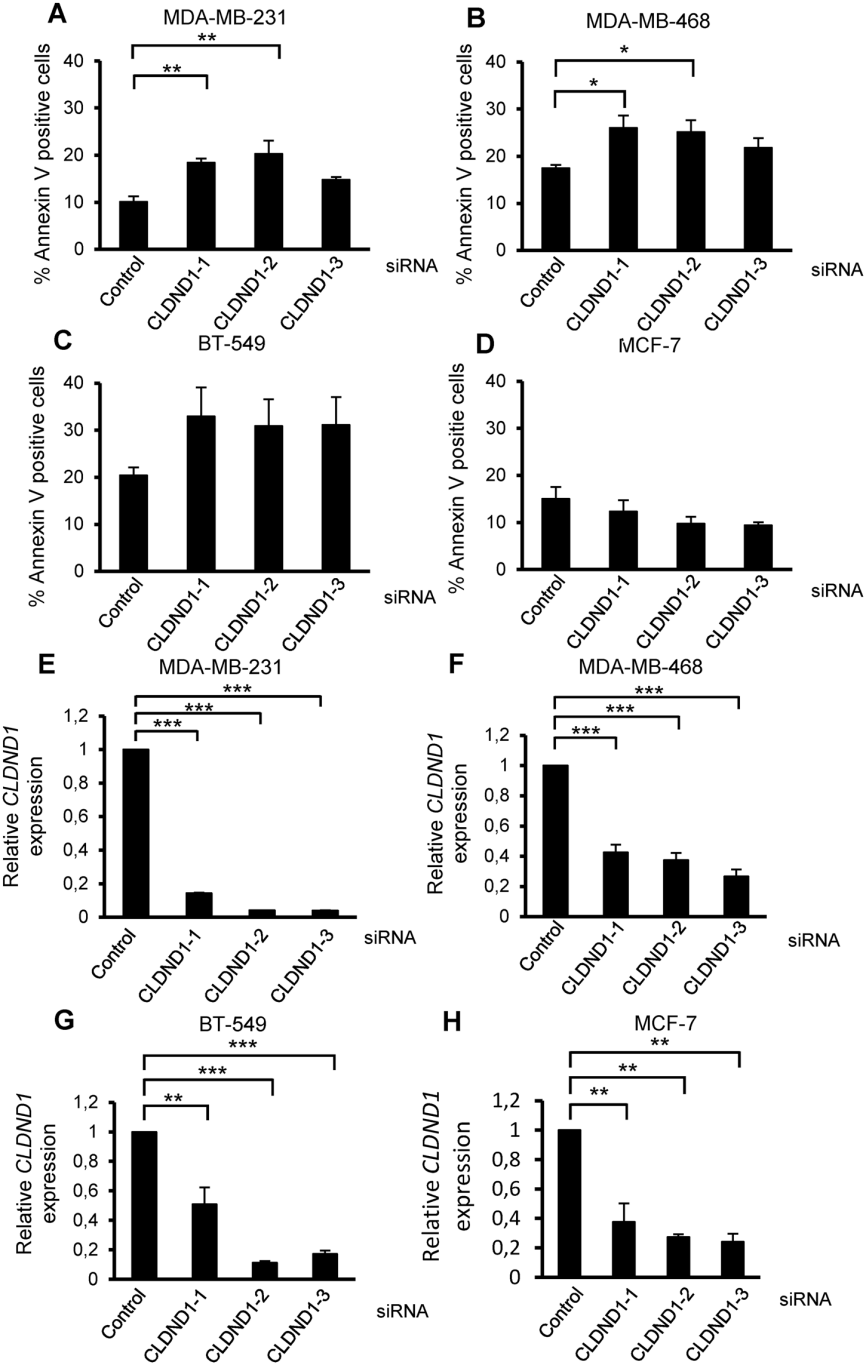
Silencing of CLDND1 induces death in various breast cancer cell lines. MDA-MB-231, MDA-MB-468, BT-549, MCF-7 cells were transfected with three different siRNA oligonucleotides targeting CLDND1 or with a control siRNA for 72 h (A-H). Cells were then either subjected to Annexin V-APC staining and flow cytometry analysis (A-D) or analyzed for mRNA expression of CLDND1 with real-time quantitative PCR (E-H). Data in A-D (mean ± S.E.M., n = 3) represent percent AnnexinV-positive cells related to control conditions and in E-F (mean ± S.E.M., n = 2–3) represent CLDND1 mRNA levels normalized to control. Asterisks indicate statistically significant differences (*, p<0.05; **, p<0.01; ***, p<0.001) compared with control cells.

To validate that the decrease in viable cell number is due to increased cell death, an Annexin-V assay was done following treatment with the siRNAs. An increase in cell death as measured with Annexin V positivity was detected with CLDND1 depletion in MDA-MB-231 and MDA-MB-468 cells (Fig 3A and 3B). It was significant for two of the siRNAs (p<0.01 in MDA-MB-231 cells and p<0.05 in MDA-MB-468 cells) and a similar tendency was observed with the third one. For BT-549 cells a tendency to increased, albeit not significant, was seen with all siRNAs (Fig 3C). However, no increase in cell death was obtained in MCF-7 cells (Fig 3D). The knockdown of CLDND1 in each of these cells was confirmed with qPCR analysis of CLDND1 gene (Fig 3E–3H).

### CLDND1 depletion induces cleavage of caspase 3 and PARP in addition to cytochrome c release from mitochondria

**Fig 4:**
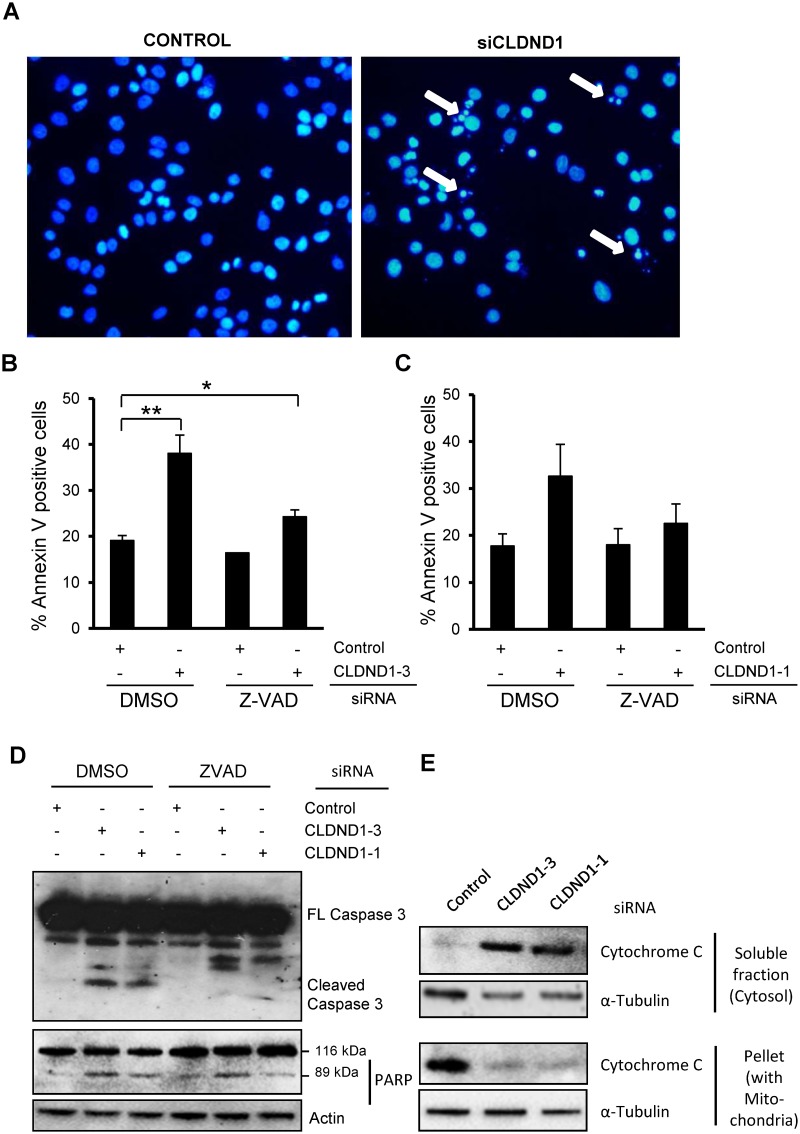
Involvement of caspase 3 and PARP cleavage together with cytochrome c release in siCLDND1 mediated effetcs. MDA-MB-468 cells following transfection with siRNA targetting CLDND1 or with a control siRNA for 72h, were either subjected to DAPI staining followed by evaluation of nuclear morphology (A), to Annexin V-APC staining and flow cytometry analysis (B, C) or harvested for Western blotting (D, E). In western blot analysis, whole cell lysates (D) or subcellular fractions (E) of the MDA-MB-468 cell lines were prepared and analyzed for caspase-3, PARP and cytochrome c. Where indicated 20 μM of z-vad or an equal volume of dimethyl sulfoxide (DMSO) was present from the initiation of transfection. Arrows in (A) indicate condensed and/or fragmented nuclei. Data in B and C (mean ± S.E.M., n = 2–3) represent percent Annexin V-positive cells related to control conditions. Western blots are representative of three independent experiments.

Since increase in Annexin V positivity does not distinguish between apoptotic and necrotic cells, we next examined the effects of CLDND1 down regulation on apoptosis in MDA-MB-468 cells. For this purpose, the nuclear morphology of the MDA-MB-468 cells was analyzed by DAPI staining (Fig 4A). Contrary to control cells (Fig 4A left), cells treated with siCLDND1 (Fig 4A right) showed apoptotic morphology characterized by several condensed or fragmented nuclei. We further evaluated the role of caspases by using the pan-caspase inhibitor zVAD-fmk (Fig 4B and 4C). Annexin V analysis revealed that zVAD-fmk suppressed cell death induced by CLDND1 depletion (p<0.05 for siCLDND1-3. In addition, down regulation of CLDND1 induced cleavage of caspase 3 and PARP (Fig 4D). The caspase 3 cleavage was abolished if cells were treated with Z-VAD-fmk. CLDND1 depletion also triggered a rapid release of cytochrome c from mitochondria (Fig 4E), as determined by immunoblot analysis using cytosolic and mitochondria containing fractions. Taken together, the data indicate that CLDND1 depletion leads to apoptosis via the intrinsic pathway.

### Role of MEK-ERK, JNK, PI3K, and AKT Signaling Pathways

**Fig 5:**
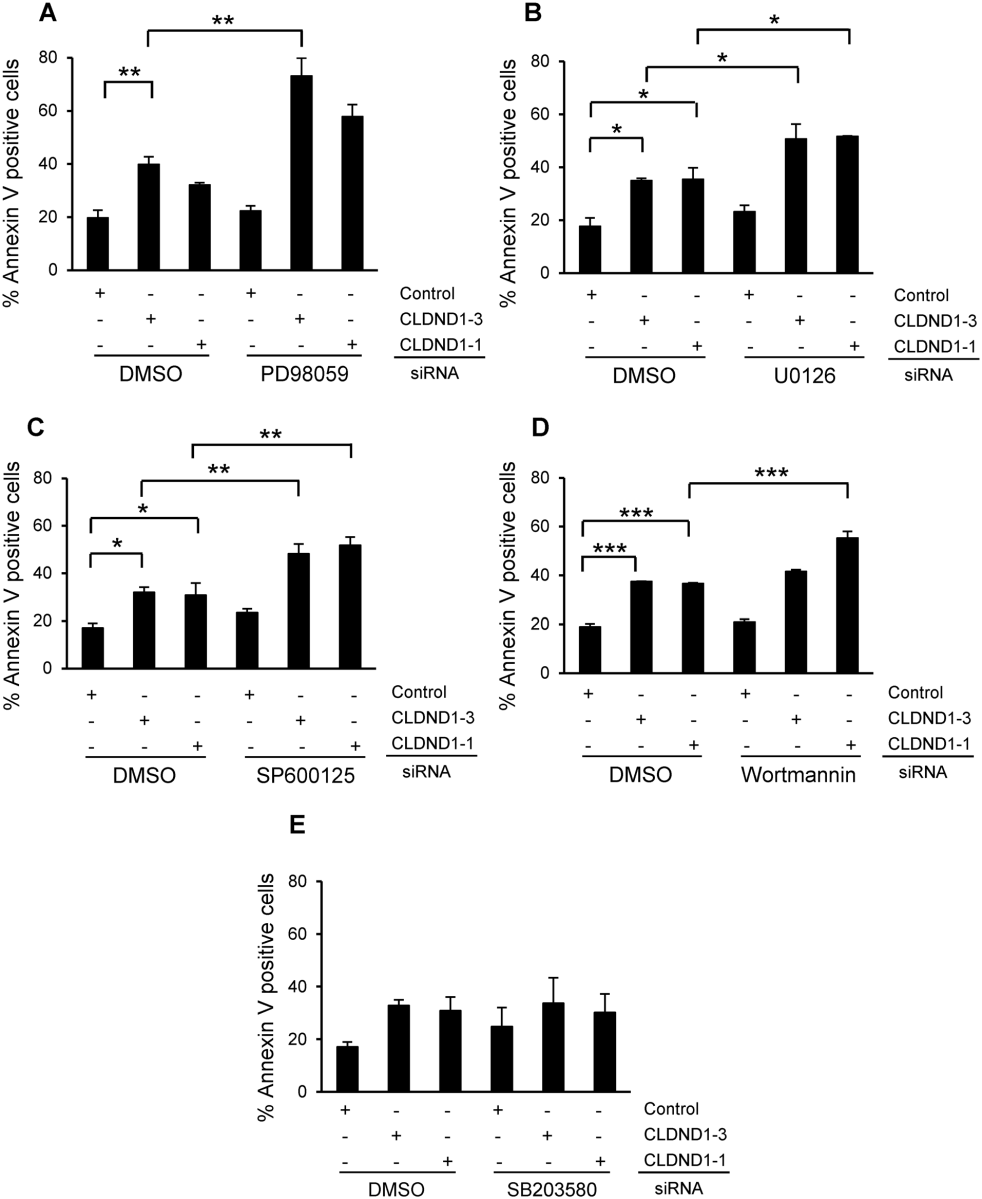
Role of MEK-ERK, JNK, PI3K, and AKT signaling pathways in CLDND1 knockdown induced cell death in MDA-MB-468 cells. MDA-MB-468 cells transfected with siRNA targeting CLDND1, or a control oligonucleotide and incubated for 72 h in complete medium before being subjected to Annexin V analysis (A-E). Where indicated, 35 μM PD98059 (A), 20 μM U0126 (B), 20 μM SP600125 (C), 0.2 μM Wortmannin (D), 10 μM SB203580(E) or an equal volume of dimethyl sulfoxide (DMSO) was present for 24h before harvesting of the cells for Annexin V analysis. Data in A–E (mean ± S.E.M., n = 2–3) show the percentage of Annexin V-positive cells related to control conditions. Asterisks indicate statistically significant differences (*, p<0.05; **, p<0.01; ***, P<0.001) compared with control cells.

Further, we sought to investigate the potential involvement of signaling mechanisms in apoptosis induced by CLDND1 down regulation. Inhibition of MEK-ERK by PD98059 (p<0.01 for siCLDND1-3) or U0126 (p<0.05 for both CLDND1 siRNAs) substantially augmented the apoptosis induced by CLDND1 down-regulation in MDA-MB-468 cells (Fig 5A and 5B). Treatment with SP600125, an inhibitor of JNK pathway exhibited a similar tendency (Fig 5C, p<0.01). The PI3K inhibitor Wortmannin also to some extent augmented cell death effects of CLDND1 depletion. On the other hand, inhibition of p38 with SB203580 did not influence the apoptotic effect of CLDND1 down-regulation in MDA-MB-468 cells (Fig 5D and 5E).

**Fig 6:**
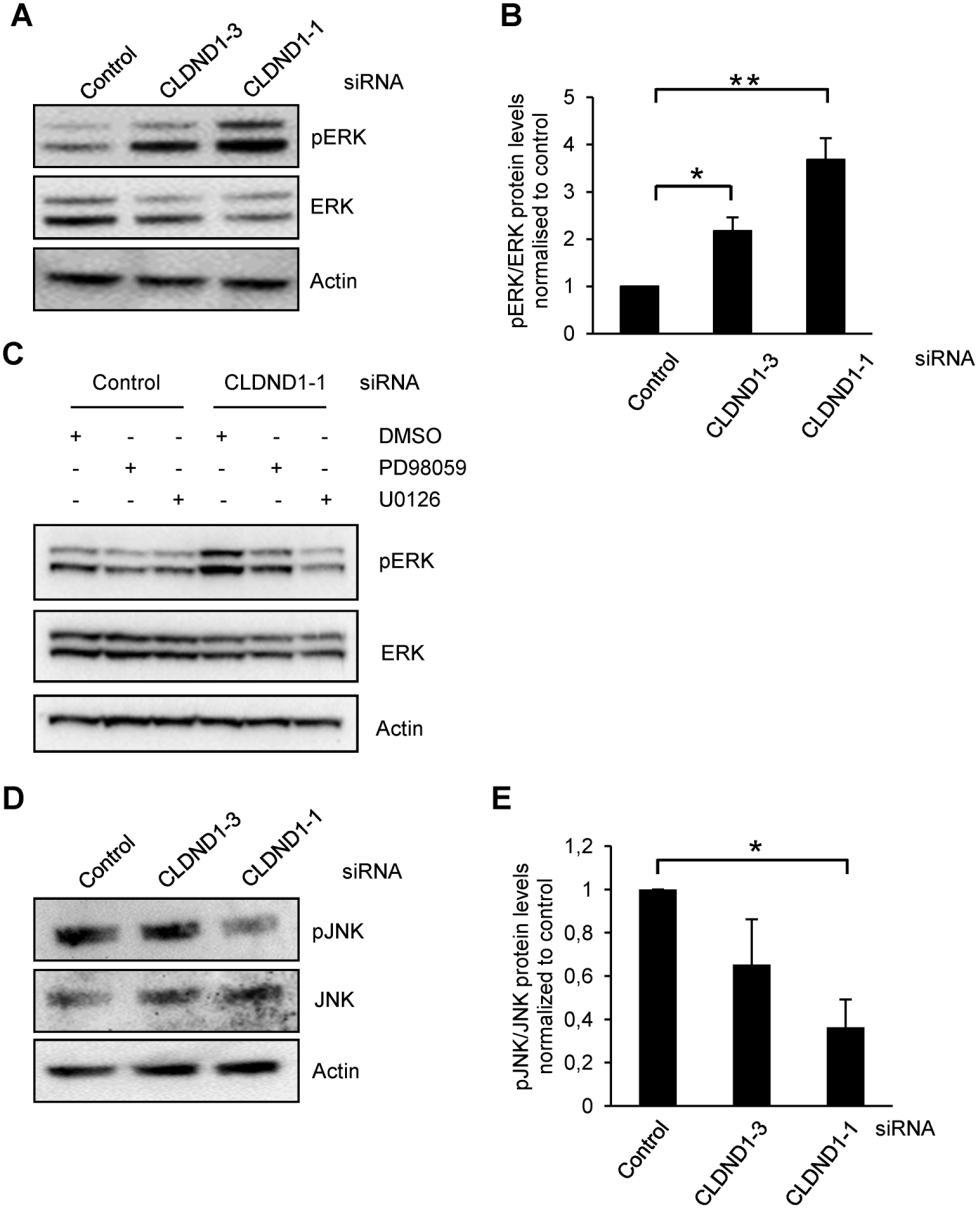
MEK-ERK inhibitor reversed siCLDND1-induced ERK activation. MDA-MB-468 cells transfected with siRNA targeting CLDND1, or a control oligonucleotide and incubated for 72 h in complete medium before being harvested for Western blotting (A, C and D). Where indicated, 35 μM PD98059, 20 μM U0126 (C) or an equal volume of dimethyl sulfoxide (DMSO) was present for 24h before harvesting of the cells for Western blotting. Whole cell lysates of the MDA-MB-468 cell line were analyzed by Western blotting (A, C and D). Data in B and E (mean ± S.E.M., n = 3) are quantifications of A and D respectively. Western blots (A, C and E) are representative of three independent experiments. Asterisks indicate statistically significant differences (*, p<0.05; **, p<0.01) compared with control cells.

Since MEK and JNK inhibitors enhanced the cell death induced by CLDND1 depletion we next checked if CLDND1 depletion has any effect on the phosphorylation of ERK and JNK. Western blot analysis revealed that CLDND1 down regulation in MDA-MB-468 cells led to a 2.1 and 3.6 fold increase in ERK1/2 phosphorylation by two different siCLDND1 oligos (Fig 6A and 6B). The MEK inhibitors PD98059 or U0126 abolished this increase (Fig 6C). Given the effects of U0126 and PD98059 on cell death in the presence of CLDND1 siRNA the increase in pERK could actually protect against an even more pronounced cell death. On the other hand, for JNK phosphorylation there was rather a suppression by siCLDND1-1 (p<0.05) (Fig 6D and 6E).

## Discussion

Accumulating evidence suggests that claudins not only play a role in epithelial and endothelial cell barrier functions but also are involved in processes of importance for malignancy, such as proliferation, invasion and metastasization. Expression of claudins has been found to be abnormal in many cancers [16–23]. Claudin expression has also been related to resistance to cell death in some tumors [27]. CLDND1 otherwise known as claudin-25 [5, 10] is a claudin-related protein whose biological functions have not been elucidated [26]. In this study we unravel a previously unknown role of CLDND1 in breast cancer cell apoptosis.

There is an accumulating amount of studies showing malignancy-promoting roles for claudins in cancer cells. Down regulation of claudin-3 was associated with reduced growth and proliferation and a significantly increased number of apoptotic cells in ovarian cancer [28]. Claudin-1 expression conferred resistance to anoikis in colon cancer cells in a Src-dependent manner [29], reduced apoposis of nasopharyngeal carcinoma cells [27] and was also found to protect MCF-7 cells from TNF-α- and tamoxifen-induced death [30, 31]. However, some claudins may instead promote cell death, exemplified by the increased chemosensitivity to cisplatin through the claudin-7-mediated up regulation of caspase pathway in human NCI-H522 lung cancer cells [32] and stimulated anchorage-independent growth promoted by silencing of claudin-6 in breast carcinoma cells [33].

Here we found that primarily the basal-like and estrogen receptor-negative MDA-MB-231 and MDA-MB-468 cells were dependent on CLDND1 for survival. The same tendency was seen in BT-549 cells, which also are basal-like. However, the estrogen receptor-positive MCF-7 cells did not depend on CLDND1 for survival. The effect may therefore be specific or more pronounced for breast cancer cells with basal-like phenotype. There were also substantial differences between the cells in CLDND1 expression, with MDA-MB-231 expressing by far the highest levels. This is also one of the most CLDND1-dependent cell lines.

CLDND1 knockdown resulted in activation of caspase 3, PARP cleavage and mitochondrial release of cytochrome c in the cytosol. This clearly indicates that the cell death is primarily through apoptosis. Likewise, down regulation of claudin-1 led to cleavage of caspase-8 and PARP, and DNA fragmentation upon TNF-α stimulation in MCF-7 cells [30]. Knockdown of claudin-1 by siRNA increased the amount of poly (ADP-ribose) polymerase (PARP) regardless of tamoxifen treatment in MCF-7 cells, but not in T47D cells [31]. Survival effects of claudins may therefore be common for breast cancer cells, but with different claudins mediating the effect depending on cell type.

It has been suggested that claudins may involve several signalling pathways. Claudin-1 has been reported to augment xenograft tumor growth and metastatic behavior in athymic mice through its effects on E-cadherin expression and β-catenin/Tcf signaling in colon carcinoma [34]. Claudin-3 and claudin-4 control tumor growth and metastases through their regulation of β-catenin signaling in ovarian cancer xenografts [35]. An important role of claudin-5 in cell motility through N-WASP and the ROCK signalling cascade has been shown in breast cancer cells [36]. Also, an enhanced invasiveness through activation of c-Abl-PKCδ signaling [37] and epithelial-mesenchymal transition through activation of the c-Abl-ERK signaling pathway [38] in response to claudin-1 has been recorded in human liver cells. Moreover, claudin-23 expression is possibly correlated with the activation of the MEK signalling pathway during pancreatic cancer cell dissociation [39]. Furthermore, claudin-7 inhibits cell migration and invasion through ERK/MAPK signalling pathway in response to growth factor stimulation in human lung cancer cells [40]. Our data rather indicate a correlation between lower CLDND1 levels and increases in ERK1/2 phosphorylation. Inhibition of the ERK1/2 pathway augmented the apoptotic effects of CLDND1 depletion, indicating that the effects of CLDND1 depletion can be further enhanced by inhibition of ERK1/2. This may therefore reflect a compensatory activation of ERK1/2 which is reminiscent of what is seen following inhibition of reactive oxygen species in Epstein-Barr Virus-positive Burkitt’s lymphoma cells [41]. We also observed that removal of CLDND1 has the opposite effect on JNK since the phosphorylation to some extent was suppressed. However, inhibition of JNK, similar to inhibition of MEK-ERK1/2 augments cell death induction following CLDND1 depletion. The effects of siCLDND1 could therefore potentially be explained by a suppression of the JNK pathway but since inhibition of JNK alone (Fig 5C) does not have a major effect on cell survival, this cannot be the sole mediator of cell death.

## Conclusions

In conclusion, we demonstrate a novel survival role for CLDND1 in breast cancer cell with basal-like features. Depletion of CLDND1 induces apoptosis and also leads to alterations in the ERK1/2 and JNK pathways. Our data provide support for a combined approach, targeting CLDND1 and other survival pathways, as a tool to elicit substantial apoptosis of breast cancer cells.
